# Higher CD27^+^CD8^+^ T Cells Percentages during Suppressive Antiretroviral Therapy Predict Greater Subsequent CD4^+^ T Cell Recovery in Treated HIV Infection

**DOI:** 10.1371/journal.pone.0084091

**Published:** 2013-12-31

**Authors:** Lillian Seu, Gabriel M. Ortiz, Lorrie Epling, Elizabeth Sinclair, Louise A. Swainson, Urmila D. Bajpai, Yong Huang, Steven G. Deeks, Peter W. Hunt, Jeffrey N. Martin, Joseph M. McCune

**Affiliations:** 1 Department of Bioengineering and Therapeutic Sciences, Department of Pharmacy, University of California San Francisco, San Francisco, California, United States of America; 2 Division of Experimental Medicine, Department of Medicine, San Francisco General Hospital, University of California San Francisco, San Francisco, California, United States of America; 3 Division of Infectious Diseases, Department of Medicine, University of California San Francisco, San Francisco, California, United States of America; 4 Core Immunology Laboratory, Department of Medicine, San Francisco General Hospital, University of California San Francisco, San Francisco, California, United States of America; 5 Division of Rheumatology, Department of Medicine, San Francisco General Hospital, University of California San Francisco, San Francisco, California, United States of America; 6 Positive Health Program, Department of Medicine, San Francisco General Hospital, University of California San Francisco, San Francisco, California, United States of America; 7 Department of Epidemiology and Biostatistics, University of California San Francisco, San Francisco, California, United States of America; Mayo Clinic, United States of America

## Abstract

HIV-mediated immune dysfunction may influence CD4^+^ T cell recovery during suppressive antiretroviral therapy (ART). We analyzed cellular biomarkers of immunological inflammation, maturation, and senescence in HIV-infected subjects on early suppressive ART. We performed longitudinal analyses of peripheral immunological biomarkers of subjects on suppressive ART (n = 24) from early treatment (median 6.4 months, interquartile range [IQR] 4.8–13.9 months) to 1–2 years of follow-up (median 19.8 months, IQR 18.3–24.6 months). We performed multivariate regression to determine which biomarkers were associated with and/or predictive of CD4^+^ T cell recovery. After adjusting for the pre-ART CD4^+^ T cell count, age, proximal CD4^+^ T cell count, and length of ART medication, the percentage of CD27^+^CD8^+^ T cells remained significantly associated with the CD4^+^ T cell recovery rate (β = 0.092 cells/ul/month, P = 0.028). In HIV-infected subjects starting suppressive ART, patients with the highest percentage of CD8^+^ T cells expressing CD27 had the greatest rate of CD4^+^ T cell recovery.

## Introduction

The hallmark of untreated HIV disease is progressive loss of CD4^+^ T cells, chronic inflammation, and generalized immune dysfunction, all leading to loss of immune control of multiple pathogens and cancers [1]. Although the initiation of suppressive antiretroviral therapy (ART) usually restores CD4^+^ T cell numbers in peripheral blood, this effect is often incomplete. Notably, suppressive antiretroviral therapy (ART) restores CD4^+^ T cell numbers in the peripheral blood but with incomplete effect: 25% of patients who start therapy with a CD4^+^ cell count of 100–200 cells/mm^3^ are unable to achieve a CD4^+^ T cell count >500 cells/mm^3^ over a mean follow-up of 7.5 years [2]. There is a growing appreciation that persistently low CD4^+^ T cell counts during treatment are associated with an increased risk of non–AIDS-related morbidities (e.g., cardiovascular disease, liver disease, and cancer) [3] and death [4]. Accordingly, many studies have recently focused on host parameters that influence optimal CD4^+^ T cell recovery or the lack thereof, documenting contributions made by variables such as host factors mediating immune activation [5], the balance between regulatory T cells and Th17 cells [6], and immune senescence [7] that influence optimal CD4^+^ T cell recovery. However, a longitudinal study that simultaneously measures a comprehensive panel of candidate immunological biomarkers in HIV subjects on early suppressive ART is lacking. Furthermore, we specifically designed our study such that the analysis of specimens occurred after the early months of successful ART suppression, upon resolution of the substantial patient-to-patient variation in the kinetics of suppression of viremia and of T cell redistribution from peripheral lymphoid tissue. Here, we have carried out such a comprehensive analysis to find that poor levels of CD4^+^ T cell recovery are predicted by high levels of CD8^+^ T cells with a senescent phenotype, i.e., increased cell surface expression of CD57 and/or decreased cell surface expression of CD27 and of CD28.

## Methods

### Ethics statement

HIV-infected adults (n = 24) on ART were recruited from the San Francisco-based SCOPE (Study of the Consequences of the Protease Era) cohort. All subjects provided written informed consent for all biologic specimens and clinical data obtained from this study. Patient informed consent forms were written in easily understandable language, and signatures were obtained and stored as described within the IRB approval. Research records were kept confidential and all biologic specimens and clinical data obtained from the study were linked to a four-digit code and not to personal identifying information. The human subjects protocol and informed consent procedure were approved by the UCSF Committee on Human Research (IRB #10-01330, reference #046371). From this cohort, we selected individuals who were treatment naïve, who started a standard ART regimen, and who had pre-ART viral loads >40,000 copies/mL (median  = 143,843, IQR 76,406–361,104 copies/ml) that declined to <1000 copies/mL after 1 month of ART (median  = 75, IQR 75–128 copies/mL). Patients experienced a median viral load decrease of 3 log_10_ copies/mL within the first month of ART. Subsequent to the specimen collection at time point 1, all subjects had documented viral loads <1000 copies/mL during the duration of this study period, with at least five recorded CD4^+^ T cell counts and five concurrently recorded HIV plasma viral load measurements of ≤1000 copies/mL (with one “blip” >1000 copies/mL permissible, as seen with patient 1006). All subjects were required to have at least five concurrently recorded HIV plasma viral load measurements and CD4^+^ T cell counts during the treatment period. Samples were obtained during suppressive ART at an early time point (TP1; median 6.4 months, IQR 4.8–13.9 months) and a later time point (TP2; median 19.8 months, IQR 18.3–24.6 months).

### Isolation of plasma and peripheral bloods mononuclear cells

Plasma and peripheral blood mononuclear cells (PBMCs) were isolated, and processed as described previously [6].

### Flow cytometry antibody labeling

The monoclonal antibodies (mAbs) used in this study were purchased from BD Biosciences (Franklin Lakes, NJ), Beckman Coulter (Indianapolis, IN), BioLegend (San Diego, CA), eBiosciences (San Diego, CA), and Invitrogen (Carlsbad, CA) (Table S1). The IL-17 cytokine assay was performed *in vitro* on cells after stimulation with Leukocyte Activation Cocktail, with BD GolgiPlug™ (BD Biosciences) and monensin (eBiosciences) at the recommended concentrations for 6 hours at 37°C. Cell preparation, cytokine detection and phenotyping were performed by previously described methods [6].

### Measurement of tryptophan and kynurenine levels in plasma

To measure IDO activity, the levels of tryptophan and kynurenine in plasma were measured by high-performance liquid chromatography coupled with tandem mass spectrometry, as described previously [6].

### Statistical analyses

**Figure 1:**
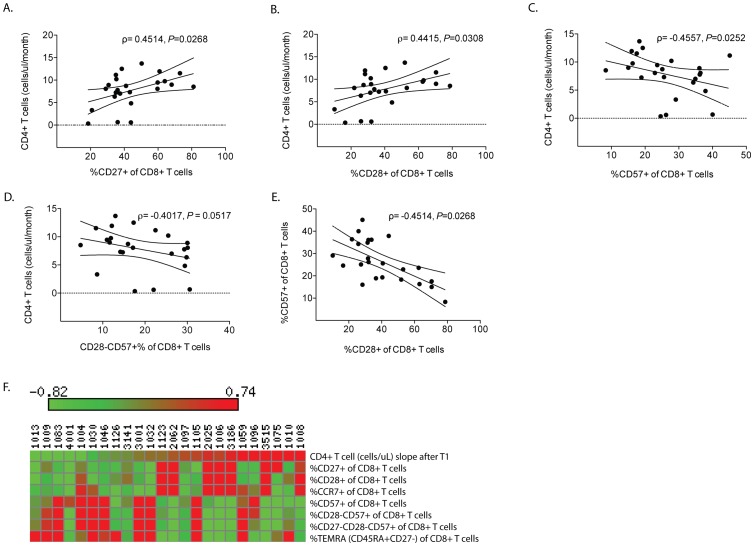
CD4^+^ T cell recovery in HIV subjects after the initiation of ART. Regression analysis of CD4^+^ T cell recovery between TP1 and TP2 and the percent of CD8^+^ T cells at TP1 expressing cell surface markers expressing (A) CD27, (B) CD28, (C) CD57, or (D) CD57 in the absence of CD28. (E) Regression analysis of the percent of CD8^+^ T cells expressing CD28 or CD57. Results are reported using the Spearman rank correlation coefficient rho (ρ). Linear regression was performed. (F) Heat map of CD8^+^ T cell surface markers according to extent of CD4^+^ T cell recovery from TP1. The scale bar represents the increasing values, and the rows of the matrix were normalized to have mean zero, variance one.

For determination of cellular expression levels by flow cytometry, the FlowJo v.9.0 program by Treestar (Ashland, OR) was used, and percent positive gates were analyzed as indicated by cellular surface marker lineage indicated in Table 1. Paired tests using STATA v11.2 (StataCorp LP, College Station, TX, USA) were performed to compare changes in levels of biomarkers over time (TP1 and TP2). Regression analyses (linear and non-linear) and Spearman correlation analyses across specified biomarkers of interest were performed using GraphPad Prism v5.0d (Graphpad Software, La Jolla, CA, USA) (Figure 1). Among subjects on ART, the β coefficient of the slope of the CD4^+^ T cell measurements was determined by measuring the linear regression of the CD4^+^ T cell from TP1 to TP2 (Figure S1). Heat maps for clinical and flow cytometry data (e.g., as shown in Figures 1F) were generated using the Matrix2png program (http://www.chibi.ubc.ca/matrix2png/). The rows of each matrix were normalized to have a mean of zero and a variance of one. Overall, we conducted tests of 33 biomarkers on a limited numbers of patients due to the stringent inclusion criteria needed for meaningful analysis. While simultaneous testing has the potential to inflate Type 1 error, multiple testing corrections necessarily inflate Type 2 error, increasing the chance of false negative conclusions- particularly when the data under evaluation are not random numbers but actual observations on nature [8]. We did not wish to err on the side of inflating the Type 2 error for this study and we accordingly did not perform multiple corrections on this analysis.

**Table 1:** Cellular expression levels (+%) of immunological biomarkers in HIV-infected subjects on suppressive ART.

		ART T1: 6.4 (4.8–13.9) months*	ART T2: 29.3 (27.3–38.9) months*	P value^a^
CD4+ T cells (cells/mm^3^)	Total (cells/uL)	300.0 (222.5–360.7)	425 (328.25–513.5)	***
Memory (+%)	Naïve (CD45RA^+^CD27^+^)	20.8 (12.8–33.6)	27.4 (21.8–40.1)	NS
	Central Memory (CD45RA^−^CD27^+^)	49.2 (41.9–55.1)	44.3 (36.0–53.4)	NS
	Effector Memory (CD45RA^−^CD27^−^)	22.7 (12.2–31.6)	13.5 (11.0–26.1)	NS
	T Effector Memory RA^+^ (CD45RA^+^CD27^−^)	4.5 (2.4–8.3)	2.7 (1.4 –8.1)	NS
Th17 and Treg (+%)	T regulatory (CD25^++^FoxP3^+^)	3.9 (3.3–5.0)	4.7 (3.5–6.3)	NS
	Th17 (IL-17^+^)	3.0 (2.3–3.8)	2.2 (1.7–2.7)	*
	Treg/Th17 ratio	0.7 (0.5–0.9)	0.5 (0.3–0.6)	NS
Senescence (+%)	CD27	72.0 (56.7–84.8)	82.3 (67.4–86.0)	NS
	CD28	92.1 (81.7–98.3)	95.8 (83.6–98.1)	NS
	CD57	7.0 (1.9–17.2)	4.4 (2.3–17.8)	NS
	CD28^−^CD57^+^	1.7 (0.4–6.0)	1.2 (0.4–6.1)	NS
Activation (+%)	PD1	27.7 (22.4–37.1)	21.4 (16.0–26.7)	**
	CD38	57.0 (50.0–68.0)	57.0 (51.8–69.8)	NS
	CD38^+^HLADR^+^	10.4 (7.3–16.6)	6.9 (4.9–11.7)	**
	CD38^+^HLADR^+^PD1^+^	5.5 (3.6–9.1)	3.2 (1.6–4.2)	**
CD8+ T cells	Total (cells/uL)	1085.0 (719.0–1382.0)	1113.5 (767.5–1590.5)	NS
Memory (+%)	Naïve (CD45RA^+^CD27^+^)	16.2 (13.1–33.9)	22.1 (13.1–36.3)	NS
	Central Memory (CD45RA^−^CD27^+^)	20.5 (18.2–25.3)	21.5 (17.4–26.9)	NS
	Effector Memory (CD45RA^−^CD27^−^)	20.1 (14.9–25.7)	15.1 (9.3–19.8)	NS
	T Effector Memory RA^+^ (CD45RA^+^CD27^−^)	35.5 (25.0–49.5)	32.1 (18.3–50.4)	NS
Senescence (+%)	CD27	39.7 (35.0–59.7)	49.2 (34.0–62.6)	NS
	CD28	32.9 (27.8–52.5)	49.5 (32.6–66.3)	NS
	CD57	25.3 (18.6–35.5)	26.6 (20.3–32.9)	NS
	CD28^−^CD57^+^	17.2 (12.0–27.8)	18.1 (10.0–26.7)	NS
Activation (+%)	PD1	24.4 (15.0–36.4)	20.4 (11.5–23.7)	*
	CD38	79.3 (70.4–83.5)	67.6 (59.8–74.0)	**
	CD38^+^HLADR^+^	41.7 (23.5–51.8)	25.2 (14.0–33.7)	**
	CD38^+^HLADR^+^PD1^+^	11.0 (6.8–20.6)	6.2 (3.3–9.6)	**
SSC++Lin- (+%)	Myeloid DCs (HLADR^+^CD11c^+^)	3.9 (2.7–5.4)	2.8 (2.3–4.4)	NS
	Plasmacytoid DCs (HLADR^−^CD11c^−^)	2.2 (1.7–2.9)	2.2 (1.4–3.3)	NS
	Classical Monocytes (HLADR^+^CD11c^+^CD14^++^CD16^−^)	70.3 (67.8–75.1)	78.8 (74.35–83.1)	**
	Non-Classical Monocytes (HLADR^+^CD11c^+^CD14^+^CD16^+^)	3.0 (1.6–6.2)	3.8 (2.3–5.7)	NS
Plasma analyte (uM)	Kyn/Tryp ratio	0.06 (0.05–0.07)	0.05 (0.04–0.07)	NS

Values reported as Median (interquartile range 1– interquartile range 3).

^a^ Analysis was performed with paired Student's T test, P value: **** <0.0001, *** <0.001, ** <0.01, * <0.05.

## Results

### Cellular expression levels of biomarkers in HIV subjects during suppressive ART

At the time of early ART suppression, subjects (n = 24) had a median age of 46 (interquartile range [IQR] 42 – 48 years) and a CD4^+^ T cell count of 300 cells/mm^3^ (IQR 222.5–360.7). Subjects were followed for a median of 29.3 months (IQR 27.3–38.9 months) thereafter. CD4^+^ and CD8^+^ T cells from an early time point (TP1) (median 6.4 months, IQR 4.8–13.9 months) and a later time point (TP2) (median 19.8 months, IQR 18.3–24.6 months) after the initiation of ART were assessed for immunological biomarkers that might plausibly play a role in HIV disease progression and/or response to treatment (Table 1), including those associated with differentiation [9], [10], effector functions [11], senescence [7], [12], activation [5], [13], [14], and anergy [15] of CD4^+^ and CD8^+^ T cells. Subpopulations of functional effector CD4^+^ T cells were defined by the expression of interleukin-17 (Th17 cells) after leukocyte activation, for the expression of FoxP3 and/or CD25 (T regulatory cells) [6], [11], and for a CD45RA^+^CD27^+^ phenotype [10]. Cells that were lineage negative and side scatter high were defined phenotypically as myeloid dendritic cells (HLADR^+^, CD11c^+^), classical monocytes (HLADR^+^, CD11c^+^, CD14^++^, CD16^−^), non-classical monocytes (HLADR^+^, CD11c^+^, CD14^+^, CD16^+^) [16], and plasmacytoid dendritic cells (CD11c^−^ and HLADR^+^) [17]. The activity of the tryptophan-catabolizing enzyme, IDO, was measured by studying the ratio of kynurenine to tryptophan in the peripheral blood [6].

Most of the immune parameters that we analyzed did not change in a given individual between TP1 and TP2. We observed no significant changes in the fractions of CD4^+^ and CD8^+^ T cells expressing markers of T cell differentiation, senescence, or discrete polarized subpopulations. There were also no significant changes in the fractions of circulating myeloid DCs, non-classical monocytes, or plasmacytoid DCs. However, we observed significant down-regulation of activated and/or anergic phenotypes (e.g., PD-1, CD38, and/or HLA-DR) of CD4^+^ and CD8^+^ T cells during suppressive ART. In addition, there was a significant decrease in the fraction of Th17 cells (P<0.05) and an increase in the frequency of classical (HLADR^+^CD11c^+^CD14^++^CD16^−^) monocytes (P<0.01). These results suggest that immune dysfunction may be partially reversed during suppressive ART-mediated viral suppression.

### Circulating levels of CD27^+^CD8^+^ T cells predict subsequent CD4^+^ T cell recovery rate

ART-treated subjects with the highest rate of CD4^+^ T cell recovery had the highest frequency of CD8^+^ T cells expressing CD27 at TP1. In particular, these T cells were visualized as having high expression of CD27 (Spearman's rho ρ = 0.45, P<0.03) (Figure 1A) and/or of CD28 (ρ = 0.44, P = 0.03) (Figure 1B), and low expression of CD57 (ρ = −0.45, P<0.03) (Figure 1C). The percentage of CD28^−^ CD57^+^ cells among CD8^+^ T cells was higher at TP1 than TP2, but only marginally predictive of poor CD4^+^ T cell recovery (ρ = −0.40, P = 0.05) (Figure 1D). After adjusting for the pre-ART CD4^+^ T cell count, age, proximal CD4^+^ T cell count, and time since ART start at TP1, the percentage of CD27^+^CD8^+^ T cells still remained significantly associated with the rate of CD4^+^ T cell recovery. A model including the above four predictors as well as the percentage of CD27^+^CD8^+^ T cells accounted for 28.5% of the variance in the CD4^+^ T cell recovery rate (cells/uL/month) (*F* (5, 18) = 2.83, P = 0.047). Most of this CD4^+^ T cell recovery rate was predicted by the percentage of CD27 expression on CD8^+^ T cells (β = 0.098 cells/uL/month, P = 0.028). Lower CD28 expression on CD8^+^ T cells at TP1 was associated with higher CD57 expression (ρ = −0.45, P<0.03) (Figure 1E). By contrast, expression of these same biomarkers on CD4^+^ T cells was not correlated with CD4^+^ T cell recovery: CD27 (ρ = −0.17, P = 0.4), CD28 (ρ = −0.046, P = 0.8), and CD57 (ρ = 0.012, P = 0.9) (data not shown). Lastly, heat map analyses taken from TP1 showed that those individuals with a low rate of CD4^+^ T cell recovery were more likely to have high percentage of CD8^+^ T cells with low levels of CD27 and CD28 and/or high levels of CD57 (Figure 1F).

## Discussion

The results of this study indicate that, while many biomarkers associated with T cell differentiation and activation were unchanged during the first several years of effective ART, those associated with CD8^+^ T cell senescence (e.g., low levels of CD27 on CD8^+^ T cells) were predictive of poor CD4^+^ T cell recovery. This finding serves to assign a rank order priority to the available immunologic biomarkers that might later guide treatment strategies to enhance immune recovery in HIV-infected subjects.

The basis for this observation may be reflective of pathways that converge to generate a state of immune senescence, one that has been described in progressive HIV disease and that results in a diminished capacity to respond to new infections and an increased frequency of non-AIDS related diseases [12]. In our comprehensive assessment of various activation, inflammation, and senescence biomarkers of CD4^+^ and CD8^+^ T cells, the strongest correlation to CD4^+^ T cell recovery was observed with alterations in the expression of CD27 on CD8^+^ T cells.

CD27 induces a co-stimulatory signal that activates NFκB, promotes survival, enhances TCR-mediated proliferative signals, and increases effector function [18]. The expression of CD27 on CD8^+^ T cells of HIV-infected subjects has been shown to promote long-term survival of functional effector–memory CD8^+^ T cells [19]. Normal human CD8^+^ T cells expanded *in vitro* have been shown to undergo replicative senescence with a progressive loss of the CD28 marker, a marker that also provides a co-stimulatory signal to T cells [20]. These expanded CD28^−^CD8^+^ T cell subsets in HIV-infected individuals display shortened telomeres [21]. CD57 has been shown to serve as a marker of diminished HIV-specific CD8^+^ T cell cytokine secretion and division [22]. In our study, CD57 expression on CD8^+^ T cells no longer predicted CD4^+^ T cell recovery after adjustment for age among other factors. Indeed, the direct role of CD57 expression in HIV pathogenesis is less clear. In recent work from our extended group, an abnormally low frequency of CD28^−^CD8^+^ T cells express CD57 in HIV infection, and persistently low CD57 on CD28^−^CD8^+^ T cells during ART appears to predict increased mortality, suggesting that CD57 expression on effector CD8^+^ T cells may be different in HIV infection than in elderly HIV-uninfected individuals [data presented at the 20th Conference on Retroviruses and Opportunistic Infections, 2013, Atlanta, GA, Abstract #309, Lee SA et al].

Our findings prompt further analysis in a larger group of ART-suppressed HIV-infected individuals to confirm whether the CD8^+^ T cell immune senescent phenotype defined by CD27, CD28, with or without CD57, is indeed predictive of CD4^+^ T cell recovery. Although the small sample size made this study underpowered to rule out potentially meaningful associations, the level of stringency needed to select a clinically relevant and homogenous patient cohort of the early ART time period necessarily led to limited recruitment numbers. We believe that this targeted approach answers a more clinically meaningful question as to what immunological biomarkers are predictive of CD4^+^ T cell recovery. Moreover, a recent study comparing PBMC transcriptomes from patients pre- and post-ART described profound changes in gene expression of various immune response genes [23]. As much of the immunological parameters of inflammation are mediated by viremia itself [14], we selected a time point in which patient specimens were available after sufficient viral suppression.

Furthermore, this study is the first of its kind to prospectively measure concurrently-derived immunological biomarkers of senescence, activation, and maturation among HIV subjects on early suppressive ART. Should it be found in such a larger study that the immune senescent phenotype defined by CD27, CD28, and/or CD57 is indeed predictive, it would then be of interest to understand the process by which these cells might arise and if these processes might be interrupted or reversed. If so, then the inclusion of such adjunctive therapies to those starting ART might lead to more robust CD4^+^ T cell recovery in a larger proportion of those who are treated. This study also calls for a better understanding of the mechanisms underlying these changes in CD8^+^ T cell function. Further research on such mechanisms might lead to treatment strategies that are more likely to result in full CD4^+^ T cell recovery in the ensuing years after early suppressive ART.

## Supporting Information

Figure S1 **CD4^+^ T cell and viral load kinetics in HIV-infected subjects on suppressive ART.** SCOPE cohort subjects (n = 24) are depicted in each graph with their 4-digit numerical ID. Subjects were followed longitudinally while on treatment to assess the slope of CD4^+^ T cell recovery that occurred between an early time point (TP1- depicted by the black “X” mark) of suppressive ART (median 6.4 months, IQR 4.8–13.9 months) to a later time point (TP2) (median 29.3 months, IQR 27.3–38.9 months). T1 was defined as the time after the administration of ART when the viral load dropped from >1000 copies/mL to <1000 copies/mL. Subsequent to TP1, all subjects had documented viral loads <1000 copies/mL during the duration of this study period, with at least five recorded CD4^+^ T cell counts and five concurrently recorded HIV plasma viral load measurements of ≤1000 copies/mL (with one “blip” >1000 copies/mL permissible, as seen with patient 1006). CD4^+^ T cell counts (cells/uL) are depicted on the left Y-axis in black, and plasma viral load measurements (p24 RNA copies/milliliter) are depicted in the right Y-axis in red in units of log_10_. The *beta* (β) coefficient of the slope of the CD4^+^ T cell measurements was determined by measuring the linear regression of the CD4^+^ T cell from T1 to T2 and is depicted on the chart. Antiretroviral therapy (ART) regimens taken during this course of follow-up are depicted on the graph as either combination (commas) or fixed dose (slash) drugs. 3-letter abbreviations for ARTs are as follows: Nucleoside reverse transcriptase inhibitors (NRTIs) [abacavir- **ABC**; didanosine- **ddI**; emtricitabine- **FTC**; lamivudine- **3TC**; stavudine- **d4T**; tenofovir- **TDF**; zidovudine -**AZT**], non-nucleoside reverse transcriptase inhibitors (NNRTIs) [efavirenz- **EFV**, nevirapine- **NVP**], Protease inhibtors (PIs) [atazanavir- **ATV**; lopinavir (with ritonavir)- **LPV**; nelfinavir- **NFV**; ritonavir- **RTV**; saquinavir- **SQV**], fusion inhibitors [enfuvirtide –**ENF**], and fixed-dose combinations [Combivir zidovudine/lamivudine- **AZT/3TC**; Trizivir zidovudine/lamivudine/abacavir- **AZT/3TC/ABC**; Epzicom abacavir/lamivudine -**ABC/3TC**; Truvada emtricitabine/tenofovir – **FTC/TDF**; Atripla efavirenz/emtricitabine/tenofovir- **EFV/FTC/TDF**].
(PDF)

Table S1 **Antibodies used for flow cytometry staining.**
(DOCX)
