# Supplementary material for: Higher CD27+CD8+ T Cells Percentages during Suppressive Antiretroviral Therapy Predict Greater Subsequent CD4+ T Cell Recovery in Treated HIV Infection
Source: PLoS One. 2013 Dec 31;8(12):e84091. doi: 10.1371/journal.pone.0084091 (PMC3877182; doi:10.1371/journal.pone.0084091)

Supplementary Figure 1: CD4+ T cell and HIV viral load kinetics in HIV-infected subjects on suppressive ART

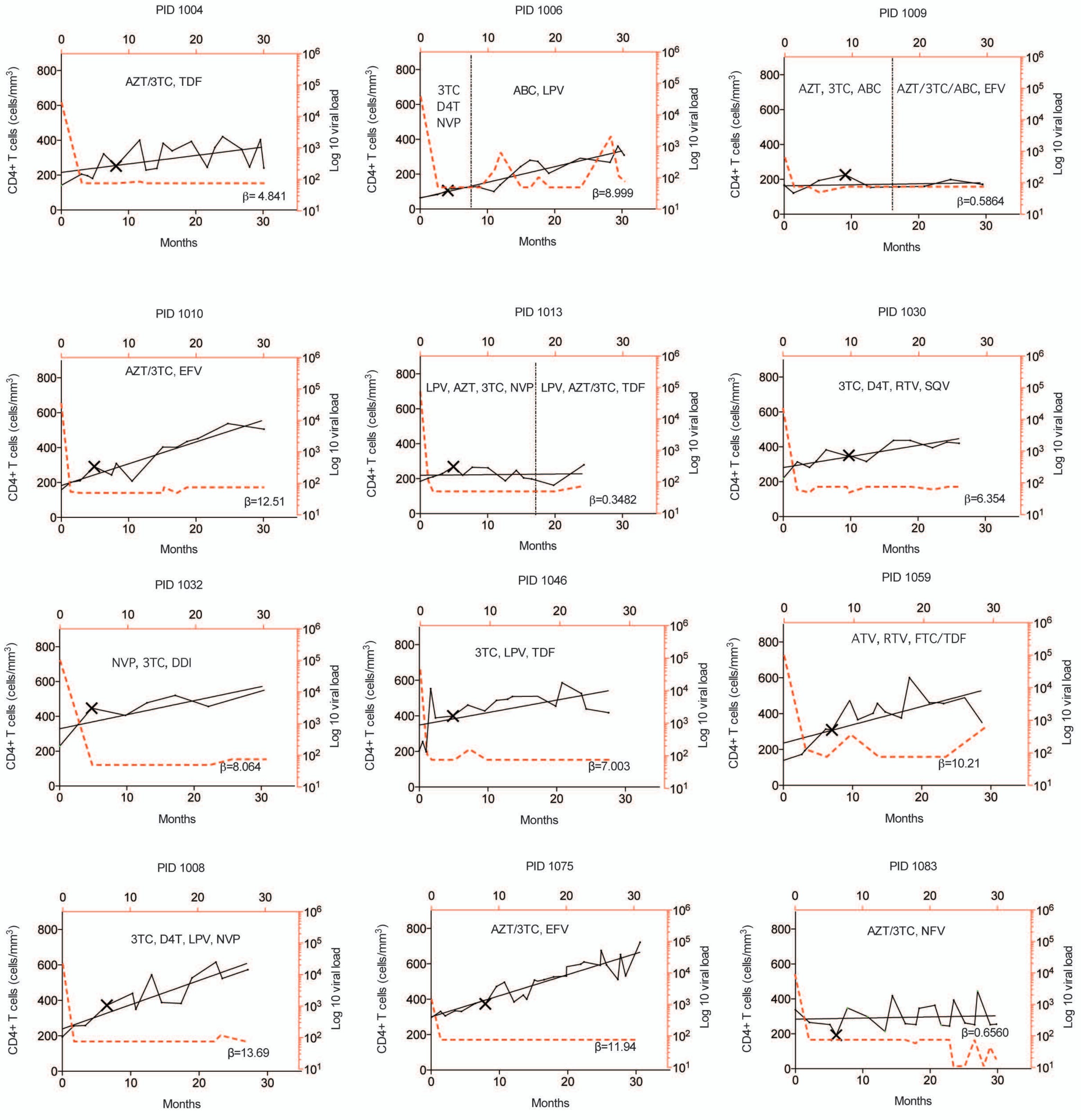

Supplementary Figure 1 (cont.)

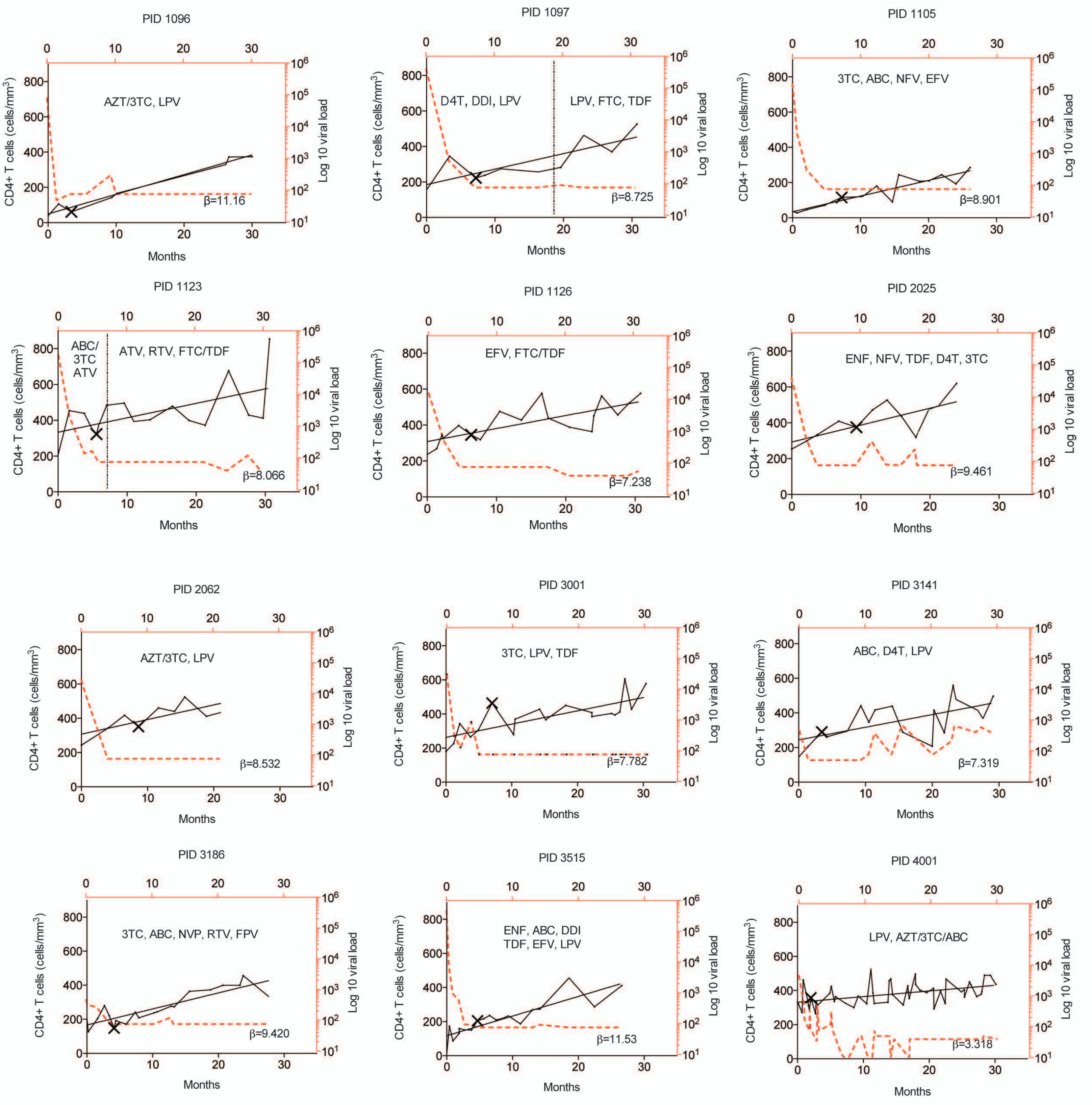

Supplement: Figure S1 — CD4+ T cell and viral load kinetics in HIV-infected subjects on suppressive ART. SCOPE cohort subjects (n = 24) are depicted in each graph with their 4-digit numerical ID. Subjects were followed longitudinally while on treatment to assess the slope of CD4+ T cell recovery that occurred between an early time point (TP1- depicted by the black “X” mark) of suppressive ART (median 6.4 months, IQR 4.8–13.9 months) to a later time point (TP2) (median 29.3 months, IQR 27.3–38.9 months). T1 was defined as the time after the administration of ART when the viral load dropped from >1000 copies/mL to <1000 copies/mL. Subsequent to TP1, all subjects had documented viral loads <1000 copies/mL during the duration of this study period, with at least five recorded CD4+ T cell counts and five concurrently recorded HIV plasma viral load measurements of ≤1000 copies/mL (with one “blip” >1000 copies/mL permissible, as seen with patient 1006). CD4+ T cell counts (cells/uL) are depicted on the left Y-axis in black, and plasma viral load measurements (p24 RNA copies/milliliter) are depicted in the right Y-axis in red in units of log10. The beta (β) coefficient of the slope of the CD4+ T cell measurements was determined by measuring the linear regression of the CD4+ T cell from T1 to T2 and is depicted on the chart. Antiretroviral therapy (ART) regimens taken during this course of follow-up are depicted on the graph as either combination (commas) or fixed dose (slash) drugs. 3-letter abbreviations for ARTs are as follows: Nucleoside reverse transcriptase inhibitors (NRTIs) [abacavir- ABC; didanosine- ddI; emtricitabine- FTC; lamivudine- 3TC; stavudine- d4T; tenofovir- TDF; zidovudine -AZT], non-nucleoside reverse transcriptase inhibitors (NNRTIs) [efavirenz- EFV, nevirapine- NVP], Protease inhibtors (PIs) [atazanavir- ATV; lopinavir (with ritonavir)- LPV; nelfinavir- NFV; ritonavir- RTV; saquinavir- SQV], fusion inhibitors [enfuvirtide –ENF], and fixed-dose combinations [Combivir zidovudine [file pone.0084091.s001.pdf]
