# Supplementary material for: Higher CD27+CD8+ T Cells Percentages during Suppressive Antiretroviral Therapy Predict Greater Subsequent CD4+ T Cell Recovery in Treated HIV Infection
Source: PLoS One. 2013 Dec 31;8(12):e84091. doi: 10.1371/journal.pone.0084091 (PMC3877182; doi:10.1371/journal.pone.0084091)
Supplement: Table S1 — Antibodies used for flow cytometry staining. (DOCX) [file pone.0084091.s002.docx]

|  |  |  |  |
| --- | --- | --- | --- |
| Antigen specificity | Clone | Conjugate(s) | Vendor |
| CD3 | SP34-2 | Pacific Blue, APC, A700 | BD Biosciences |
| CD4 | S3.5 | PE/Texas Red, Qdot605 | Invitrogen |
| CD8 | 3B5 | PeCy5.5, Qdot605, Qdot705 | Invitrogen |
| CD11c | B-LY6 | V450 | BD Biosciences |
| CD14 | RMO52 | ECD | Beckman Coulter |
| CD16 | 3G8 | Alexa700, APC | Invitrogen |
| CD19 | HIB19.1 | Alexa700, APC | BD Biosciences |
| CD25 | M-A251 | APC-Cy7 | BD Biosciences |
| CD27 | O323 | APC-eFluor 780 | eBioscience |
| CD28 | CD28.2 | Pecy5 | BD Biosciences |
| CD31 | WM59 | FITC | BD Biosciences |
| CD38 | HB7 | PE | BD Biosciences |
| CD45RA | L48 | PE, PECy7 | BD Biosciences |
| CD56 | NCAM16.2 | FITC, APC | BD Biosciences |
| CD57 | HCD57 | A647 | BioLegend |
| CD197 (CCR7) | 3D12 | APC-eFluor 780 | eBiosciences |
| CD279 (PD-1) | EH12.2H7 | Alexa 647 | BioLegend |
| HLA-DR | L243 | FITC, APC-Cy7 | BD Biosciences |
| IL-17 | ebio64CAP17 | Alexa647 | eBioScience |
| FoxP3 | PCH101 | Pacific Blue | eBiosciences |
